# lncRScan-SVM: A Tool for Predicting Long Non-Coding RNAs Using Support Vector Machine

**DOI:** 10.1371/journal.pone.0139654

**Published:** 2015-10-05

**Authors:** Lei Sun, Hui Liu, Lin Zhang, Jia Meng

**Affiliations:** 1 School of Information Engineering, Yangzhou University, Yangzhou, Jiangsu Province, China; 2 Jiangsu Co-innovation Center for Prevention and Control of Important Animal Infectious Diseases and Zoonoses, Yangzhou University, Yangzhou, Jiangsu Province, China; 3 School of Information and Electrical Engineering, China University of Mining and Technology, Xuzhou, JiangSu Province, China; 4 Department of Biological Sciences, Xi’an Jiaotong-Liverpool University, Suzhou, Jiangsu Province, China; CSIR-Institute of Microbial Technology, INDIA

## Abstract

Functional long non-coding RNAs (lncRNAs) have been bringing novel insight into biological study, however it is still not trivial to accurately distinguish the lncRNA transcripts (LNCTs) from the protein coding ones (PCTs). As various information and data about lncRNAs are preserved by previous studies, it is appealing to develop novel methods to identify the lncRNAs more accurately. Our method lncRScan-SVM aims at classifying PCTs and LNCTs using support vector machine (SVM). The gold-standard datasets for lncRScan-SVM model training, lncRNA prediction and method comparison were constructed according to the GENCODE gene annotations of human and mouse respectively. By integrating features derived from gene structure, transcript sequence, potential codon sequence and conservation, lncRScan-SVM outperforms other approaches, which is evaluated by several criteria such as sensitivity, specificity, accuracy, Matthews correlation coefficient (MCC) and area under curve (AUC). In addition, several known human lncRNA datasets were assessed using lncRScan-SVM. LncRScan-SVM is an efficient tool for predicting the lncRNAs, and it is quite useful for current lncRNA study.

## Introduction

Recently tens of thousands of long non-coding RNAs (lncRNAs) have been discovered in the transcriptome using biotechnology such as cDNA cloning [1, 2], tiling mircoarray [3–5] and high throughput sequencing [6, 7]. Studies also reveal that the lncRNAs are extensively involved numerous cellular processes, such as embryonic stem cell (ESC) pluripotency, erythropoiesis, cell-cycle regulation and diseases [8–11]. However, current lncRNA function studies can be hampered by lack of complete and high-quality lncRNA gene annotations, especially when conducting analysis on the genome scale. Although there appear several lncRNA data sources, such as lncRNAdb [12], NONCODE [13] and GENCODE [14], they seldom perform well-matched intersection between each other [15], which implies that the lncRNA catalogue needs to be developed. Meanwhile, with the widespread usage of deep sequencing in life science, more and more novel lncRNAs can be found due to their tissue-specific expression characteristic. The newly-discovered lncRNAs are always compared with previous annotations for guaranteeing the quality of further analysis [8, 9].

Either for lncRNA gene annotation or novel lncRNA discovery, it is crucial to evaluate the protein coding potential of a transcript. As similar as protein-coding genes, lncRNAs are RNA polymerase II products, and can be capped and polyadenylated [16], and also present similar histone-modification profiles, splicing signals and exon/intron lengths [15]. Due to the similarities between mRNAs and lncRNAs, it is challenging to separate the lncRNA transcripts (LNCTs) from the protein coding ones (PCTs) [17].

Thanks to the advance of bioinformatics, discriminating LNCTs from PCTs can be modelled as a binary classification problem, which has been solved by several computational methods, such as CONC [18], CPC [19], CNCI [20], iSeeRNA [21] and RNAcon [22]. CONC integrates various features in its classification model, but it is slow for large datasets and also performs less accurate than other newer methods such as CPC [19]. CPC uses features derived from open reading frame (ORF) and sequence alignment, and the developers also provide users with a web interface. CNCI distinguishes protein-coding and non-coding sequences by profiling adjoining nucleotide triplets, however it cannot work on large datasets. iSeeRNA outperforms previous methods and it also provides users with a program for training a new classification model based on custom dataset. RNAcon applies features of tri-nucleotide composition to the classification, but it does not show better performance than other methods in our experiment. Compared to these support vector machine (SVM) based methods inspecting the entire transcript, a comparative genomics method named PhyloCSF [23] focuses on classifying protein-coding and non-coding regions, and it has been frequently used for lncRNA identification [7, 24]. In addition, CPAT [25] is another tool for assessing coding potential of a transcript using an alignment-free logistic regression model. Based on these computational methods, lncRNA function studies usually build a pipeline to obtain a set of confident lncRNAs [16, 24, 26]. For example, a simple pipeline consisting of length filtering (> 200*nt*), CPC and BLAST [27] can make a stringent lncRNA dataset. However, it is still not convenient to master such non-standard filtering workflow.

As various information and data about lncRNAs are preserved by previous studies, it is appealing to develop new methods to identify the lncRNAs more accurately. By integrating features derived from gene structure, transcript sequence, potential codon sequence and conservation, a novel computational method is proposed here for solving the problem.

## Methods

### Gold-standard datasets

Since a reliable dataset is of importance to model training and testing, we adopted the high-quality manually-curated GENCODE gene annotations (version 19/v19 of human and version M2/vM2 of mouse) [14, 15] for constructing the gold-standard datasets, which are composed of gene annotations in **G**ene **T**ransfer **F**ormat (GTF) and corresponding genome sequences in FASTA format for human and mouse respectively. Specifically, the human gene annotation (GENCODEv19) includes 81814 PCTs and 23898 LNCTs, while the counts are 47394 and 6053 respectively for mouse (GENCODEvM2). In addition, the genome sequences of human (GRCh37/hg19) and mouse (GRCm38/mm10) were downloaded from University of California Santa Cruz (UCSC) genome browser respectively [28]. For model training and testing, we created Training-A, Testing-A and Testing-B (S1 Dataset) by splitting the gold-standard datasets (See Table 1). All transcripts in training and testing sets were randomly sampled from the complete dataset, and there are no overlapping areas between the training and testing sets. Since our prediction model was trained on Training-A, in which a proportion of transcripts may have similar sequences to that in Testing-A, which could lead to an unfair comparison between our method and the others though our method does not only use sequence features. To make a fairer comparison, Testing-B datasets were created by removing similar transcripts from Testing-A using CD-HIT (v4.6.1, cut-off 0.8) [29], compared to Training-A sequences. To alleviate the effect of imbalanced-classes, we constructed the training and testing sets with equal or similar sizes.

**Table 1:** Dataset partition.

	Traing-A	Testing set		Remaining	Sum
		Testing-A	Testing-B		
human PCTs(Positive)	5000	10000	8307	66814	81814
human LNCTs(Negative)	5000	10000	8875	8898	23898
mouse PCTs(Positive)	2500	3500	3130	41394	47394
mouse LNCTs(Negative)	2500	3500	2975	53	6053

The gold-standard datasets were divided for model training and testing. The first two rows show counts of divided human dataset while the other two ones show that of mouse. For human, Training-A includes 5000 PCTs randomly sampled from the totalling 81814 ones, and 5000 LNCTs sampled from the totalling 23898 ones. Human Testing-A was created by sampling another 10000 PCTs and LNCTs respectively, and human Testing-B containing 8307 PCTs and 8875 LNCTs was created by removing transcripts that show similar sequences (cutoff 0.8 of CD-HIT) to that in Training-A from Testing-A. Similarly for mouse, the counts of PCTs and LNCTs in Training-A are both 2500, and the count is 3500 in Testing-A. And counts of Testing-B for mouse PCTs and LNCTs are 3130 and 2975 respectively. Besides, the remaining transcripts (Remaining) were not taken into account in the analysis.

### Feature selection

Since each feature selected can affect the overall classification performance, we conducted feature selection (FS) (See S1 File) based on a set of candidate features, which were derived from our current knowledge about lncRNAs. The candidate features can be classified into three categories. The first category includes features extracted from nucleotide sequences, and they are 14 tri-nucleotides attributes, namely ACG, CCG, CGA, CGC, CGG, CGT, CTA, GCG, GGG, GTA, TAA, TAC, TAG and TCG [22], and standard deviation of stop codons (TAG, TAA and TGA) between three frames, and GC content. The second category was extracted from the output of a program called *txCdsPredict* from UCSC, which is used to predict a codon sequence with the most likelihood from an input nucleotide sequence, and it includes features like *txCdsPredict* score, CDS length and CDS percentage (CDS_length divided by transcript_length) [21]. The third category of features was extracted from gene structure of the transcript, and they are transcript length, exon count and average exon length.

Then six features (See Table 2) were selected from the total candidates by FS. First, transcript length was selected since the length of PCTs and LNCTs can be differentially distributed [7, 15, 24]. Second, with an assumption that a true PCT may have a long ORF in one of the three frames translated, we presume that the stop codons in the frame where the ORF emerges are fewer than that randomly appear in the other two frames. Therefore, we selected the standard deviation (*SCS*) of stop codon counts (*SCC*) between three frames translated as the second feature since the standard deviation of PCT should be smaller than that of LNCT (See Eqs (1) and (2)).

$$\begin{array}{c} \bar{x}=\frac{1}{3}\sum_{i=0}^{2}SCC_{i} \end{array}$$ (1)

$$\begin{array}{c} SCS=\sqrt{\frac{1}{3}\sum_{i=0}^{2}{\left(SCC_{i}-\bar{x}\right)}^{2}} \end{array}$$ (2)

**Table 2:** Selected features.

**Feature**	**Description**
transcript length	sum of exon lengths of a transcript
stop codon std	standard deviation of stop codon counts between three translated frames
CDS score	score of *txCdsPredict* prediction
exon count	exon count of a gene
exon length	average exon length of a gene
consv	average PhastCons scores of a transcript

This table lists features of lncRScan-SVM.

where $\bar{x}$ denotes the mean of stop codon counts of three frames (*SCC*_0_, *SCC*_1_ and *SCC*_2_). Then *SCS* can be calculated using Eq (2). Similarly, since the PCT may have ORF potentially, we used *txCdsPredict* to predict the codon sequence (CDS) of the candidate transcript, and the score output by *txCdsPredict* was selected as the third feature. By analysing the gene structures of transcripts, the exon count and average exon length were selected as features because the lncRNA genes may have fewer exons and be shorter than the protein-coding ones. The last feature we selected is the conservation score calculated by averaging PhastCons scores of the transcript.

### lncRScan-SVM

**Fig 1:**
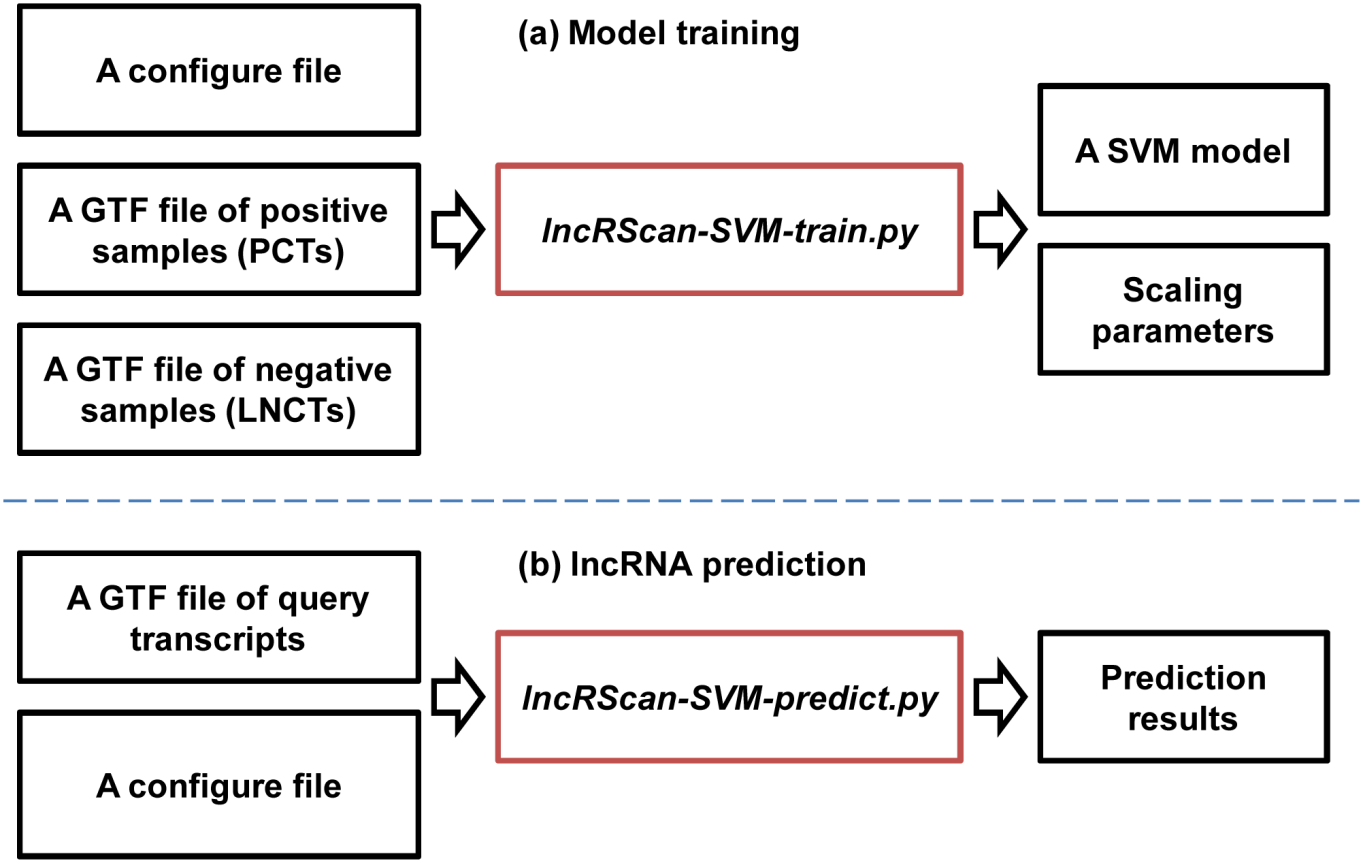
lncRScan-SVM. (a) Model training. A GTF file of positive samples, a GTF file of negative samples and a configure file are input to *lncRScan-SVM-train.py* for training a SVM model and getting scaling parameters. (b) lncRNA prediction. A GTF file of query transcripts and a configure file are input to *lncRScan-SVM-predict.py* for predicting PCTs or LNCTs.

The six features selected above were fed into the SVM framework to build a tool named lncRScan-SVM (v1.0.0), which also depends on several third-part packages/programs such as BioPython [30], LIBSVM [31] and *txCdsPredict*. Of the programs packaged by lncRScan-SVM, two scripts can commonly be used. One is *lncRScan-SVM-train.py* for training a SVM model and the other one is *lncRScan-SVM-predict.py* for predicting PCTs/LNCTs (See Fig 1). The lncRScan-SVM package is freely available on *http://sourceforge.net/projects/lncrscansvm/?source=directory*.

#### Model training

A SVM model for prediction can be generated using *lncRScan-SVM-train.py* (See Fig 1a). Specifically, the *lncRScan-SVM-train.py* program first extracts nucleotide sequences according to the gene models of training samples. Then a script *extract_features.py* is used to extract transcript features. After transferring features to a standard format of LIBSVM by *feature2svm.py*, a program *svm-scale* is used to scale the features. Finally, a prediction model can be generated.

#### lncRNA prediction

Users can use *lncRScan-SVM-predict.py* to predict LNCTs or PCTs (See Fig 1b). Specifically, the main input of *lncRScan-SVM-predict.py* is a GTF file, which lists all transcripts for classification. Then the sequence of the query transcript is extracted using *gffread* [6]. After feature extracting, formatting and scaling, *svm-predict* classifies PCTs and LNCTs using the prediction model trained previously. And the prediction models for hg19 and mm10 have been packaged in lncRScan-SVM.

### Evaluation

The performance of lncRScan-SVM is compared to other methods, such as CPC (v0.9-r2), CPAT (v1.2.2), iSeeRNA (v1.2.1) and RNAcon (v1.0), by several criteria, such as sensitivity (SES) or true positive rate (TPR) or recall, specificity (SPC), accuracy (ACC), Matthews correlation coefficient (MCC).

$$\begin{array}{c} SES=\frac{TP}{P}=\frac{TP}{TP+FN} \end{array}$$ (3)

$$\begin{array}{c} SPC=\frac{TN}{N}=\frac{TN}{TN+FP} \end{array}$$ (4)

$$\begin{array}{c} ACC=\frac{TP+TN}{P+N} \end{array}$$ (5)

$$\begin{array}{c} MCC=\frac{TP\times TN-FP\times FN}{\sqrt{\left(TP+FP\right)\times \left(TP+FN\right)\times \left(TN+FP\right)\times \left(TN+FN\right)}} \end{array}$$ (6)

where *TP*, *P*, *TN*, *N*, *FP*, *FN* denote true positive, positive, true negative, negative, false positive and false negative respectively. In this paper, ‘positive’ and ‘negative’ correspond to PCT and LNCT respectively. And area under curve (AUC) of receiver operation characteristic (ROC) are also used as another indicator [32]. In addition, PhyloCSF is not took into account as it focuses on distinguishing protein-coding regions from non-coding ones, which is slightly different from the objective of the methods for classifying PCTs and LNCTs.

### Ethics Statement

N/A

## Results and Discussion

### Performance of single feature

**Fig 2:**
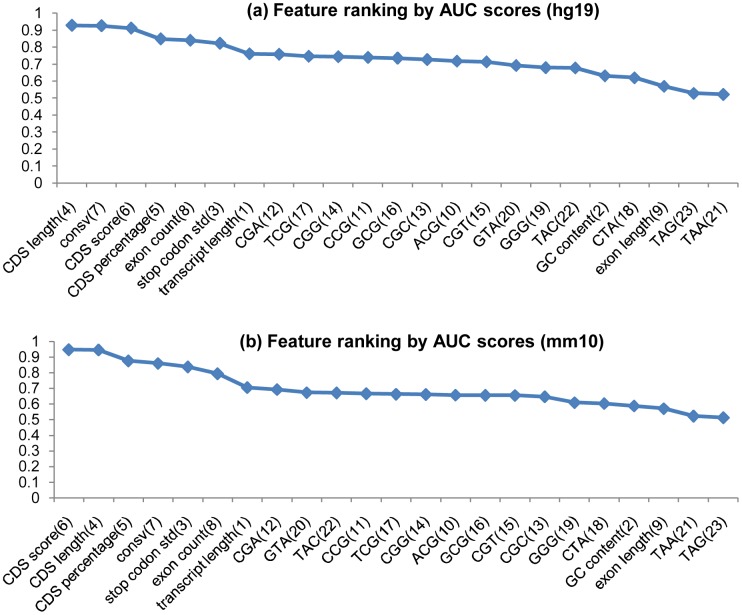
Feature ranking by AUC scores. (a) The candidate features are ranked by AUC scores calculated on hg19 Training-A; (b) The candidate features are ranked by AUC scores calculated on mm10 Training-A. Each feature ID is labelled in brackets.

Since every candidate feature can contribute to the overall classification performance of a predictor, the performance of each feature was evaluated by AUC scores on the hg19 and mm10 Training-A datasets respectively (See Fig 2). As seen from the ranking result, the top eight features are CDS length, consv, CDS score, CDS percentage, exon count, stop codon std, transcript length and CGA, which show slightly different orders between hg19 and mm10. Particularly, five of the six lncRScan-SVM features, namely transcript length, stop codon std, CDS score, exon count and consv, are included in the top eight ranked features, which indicates that most of the lncRScan-SVM features can contribute positively to the predictor. In addition, the features of hg19 and mm10 represent similar performance as seen from the the Pearson correlation coefficient (0.9484604) of the AUC scores, which shows the robustness of classification performance of the candidate features between the species.

### Comparison of prediction methods

The prediction performance of lncRScan-SVM was evaluated by comparing to several other methods, such as CPC, CPAT, iSeeRNA and RNAcon, on Testing-A and Testing-B. When testing iSeeRNA, we used two kinds of models. One is the default iSeeRNA prediction model and the other one (iSeeRNA2) is generated by re-training iSeeRNA on Training-A. As a result, lncRScan-SVM outperforms other methods in most aspects, such as SPC, ACC, MCC and AUC (See Tables 3 and 4).

**Table 3:** Method comparison on Testing-A and Testing-B of hg19.

	Testing-A					Testing-B				
	SES(%)	SPC(%)	ACC(%)	MCC(%)	AUC(%)	SES(%)	SPC(%)	ACC(%)	MCC(%)	AUC(%)
CPC	**97.62**	67.23	82.425	68.069	94.55	**97.328**	69.262	82.831	68.898	94.78
CPAT	85.28	**94.60**	89.94	80.23	95.17	83.941	**95.223**	89.768	79.896	95.14
*lncRScan-SVM*	89.20	93.88	**91.54**	**83.17**	**96.39**	88.215	94.479	**91.45**	**82.985**	**96.39**
iSeeRNA	87.97	92.32	90.13	80.36	95.33	87.04	92.965	90.082	80.238	95.28
iSeeRNA2	90.02	92.409	91.205	82.44	96.23	89.103	92.885	91.045	82.106	96.18
RNAcon	69.11	84.53	76.82	54.29	86.11	68.039	85.454	77.034	54.475	86.1

CPC and CPAT were run by submitting the GTF files of Testing-A and Testing-B through their web interfaces. The lncRScan-SVM predictor was run after feature scaling. Besides, we tested the default iSeeRNA predictor and iSeeRNA2, an iSeeRNA model re-trained on Training-A, as well as RNAcon with a parameter *T* equals 0. The biggest value in each column is in bold font while the smallest one is underlined.

**Table 4:** Method comparison on Testing-A and Testing-B of mm10.

	Testing-A					Testing-B				
	SES(%)	SPC(%)	ACC(%)	MCC(%)	AUC(%)	SES(%)	SPC(%)	ACC(%)	MCC(%)	AUC(%)
CPC	**98.371**	75.457	86.914	75.847	95.43	**98.211**	78.42	88.567	78.483	95.75
CPAT	88.171	**95.343**	91.757	83.73	96.38	87.891	95.966	91.826	83.979	96.46
*lncRScan-SVM*	89.143	95.286	**92.214**	**84.588**	**96.62**	88.53	**96.067**	**92.203**	**84.693**	**96.64**
iSeeRNA	85.411	93.126	89.266	78.768	95.26	85.517	93.739	89.521	79.379	95.51
iSeeRNA2	88.426	93.27	90.846	81.79	95.98	87.958	93.807	90.806	81.793	96.04
RNAcon	73.086	79.60	76.343	52.798	84.84	72.62	80.874	76.642	53.604	85.19

The description for mm10 is the same as Table 3.

As seen from the testing result on Testing-A for either hg19 or mm10, lncRScan-SVM shows slightly smaller SPC, but higher SES and overall ACC, MCC and AUC scores than CPAT. In contrast, RNAcon presents the worst performance as it obtains the lowest score in most aspects. CPC obtains the highest SES (97.62% for hg19 and 98.371% for mm10), but it shows poor performance in other aspects, e.g. the lowest SPC score (67.23% for hg19 and 75.457% for mm10), which means CPC may ignore a considerable proportion of true lncRNAs. By training on Training-A, iSeeRNA2 shows better performance than the default one, but it is still less accurate than lncRScan-SVM.

**Fig 3:**
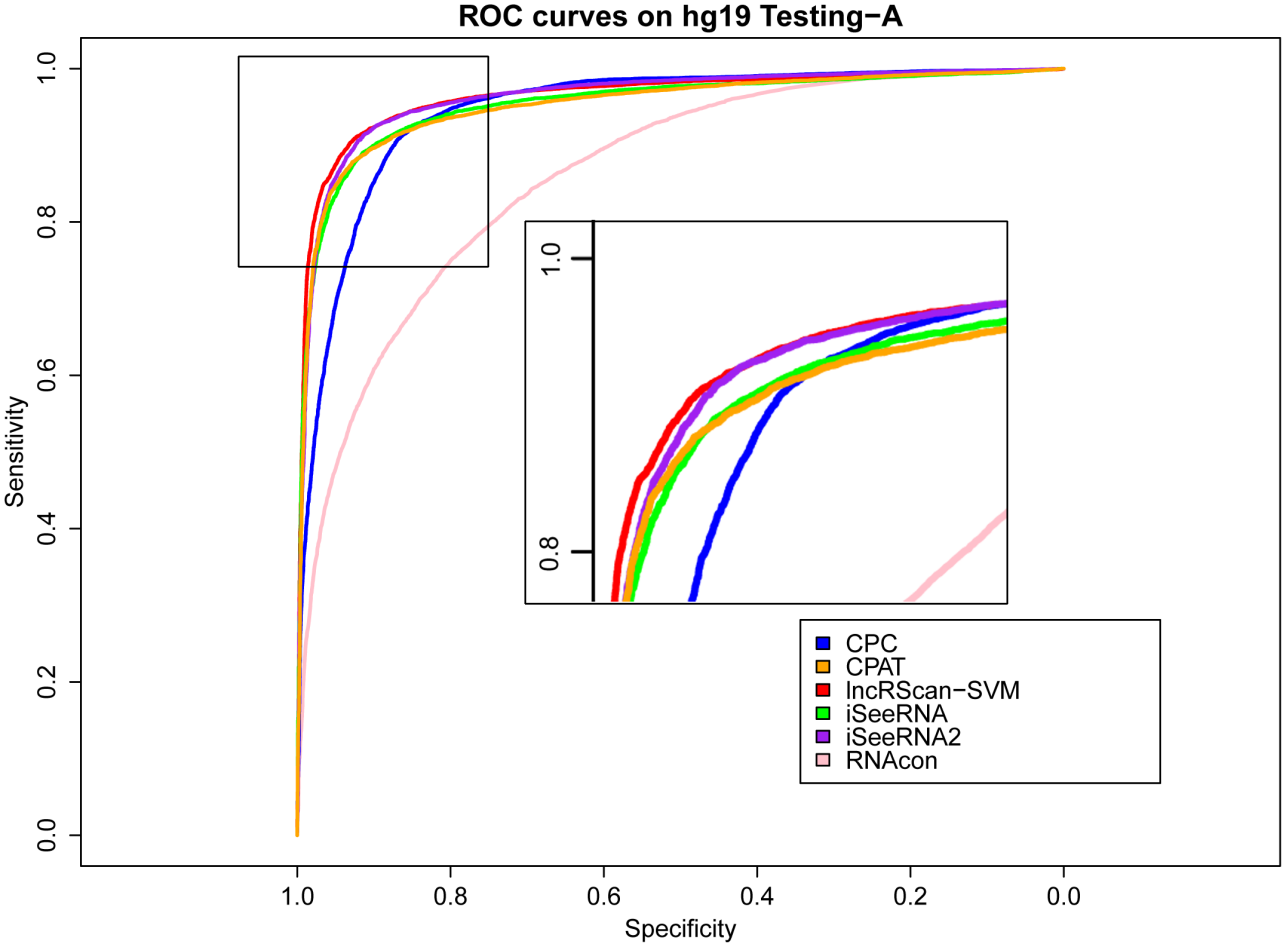
ROC curves of lncRNA prediction on hg19 Testing-A. The prediction performance of CPC, CPAT, lncRScan-SVM, iSeeRNA, iSeeRNA2 and RNAcon on hg19 Testing-A is illustrated by ROC curves with colors of blue, orange, red, green, purple and pink respectively. The definitions of the sensitivity for x axis and specificity for y axis are the same as Formulas 3 and 4. The top-left area is zoomed in on for distinguishable observation.

**Fig 4:**
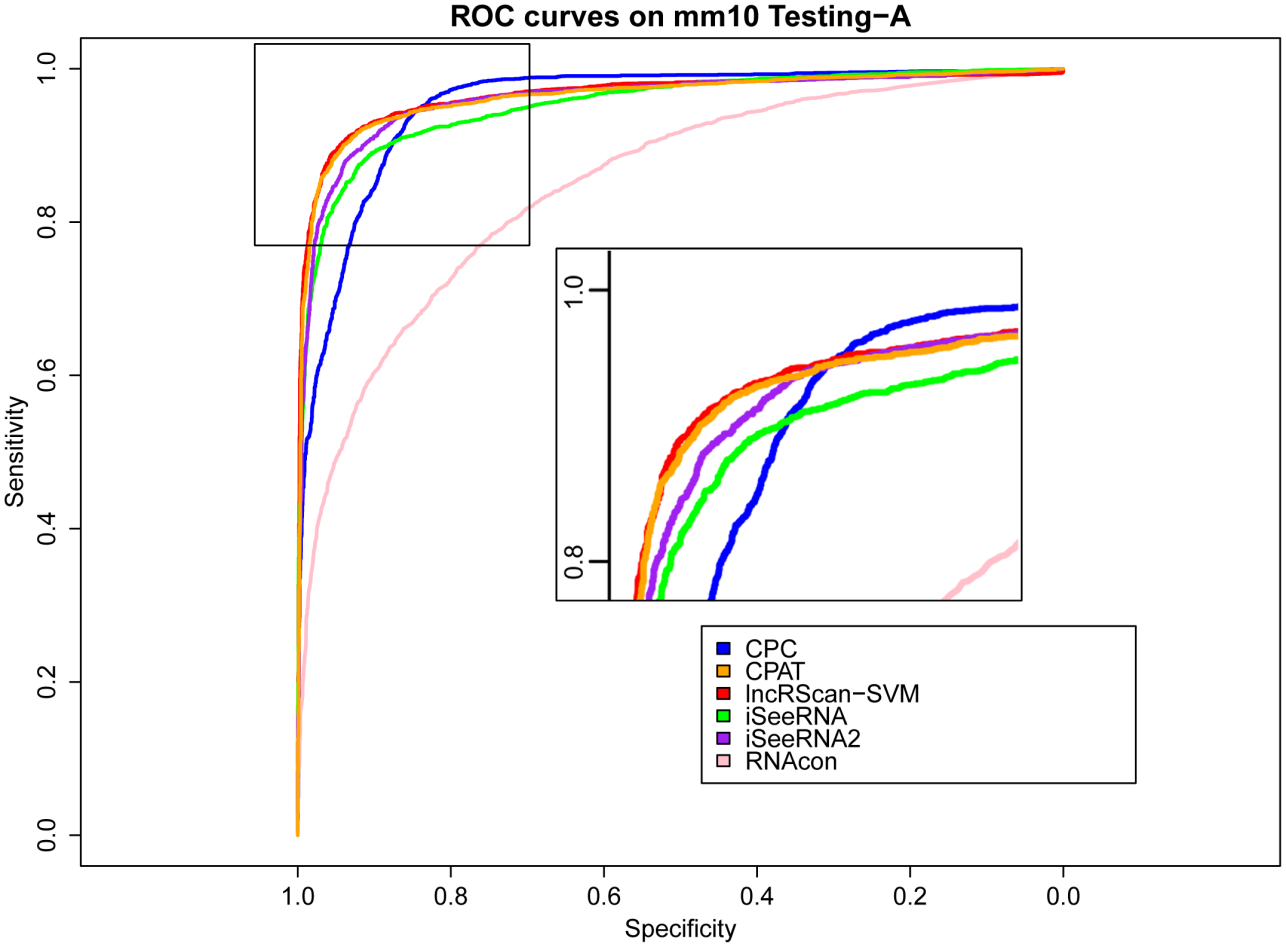
ROC curves of lncRNA prediction on mm10 Testing-A. The description of ROC curves for tests on mm10 Testing-A is the same as Fig 3.

**Fig 5:**
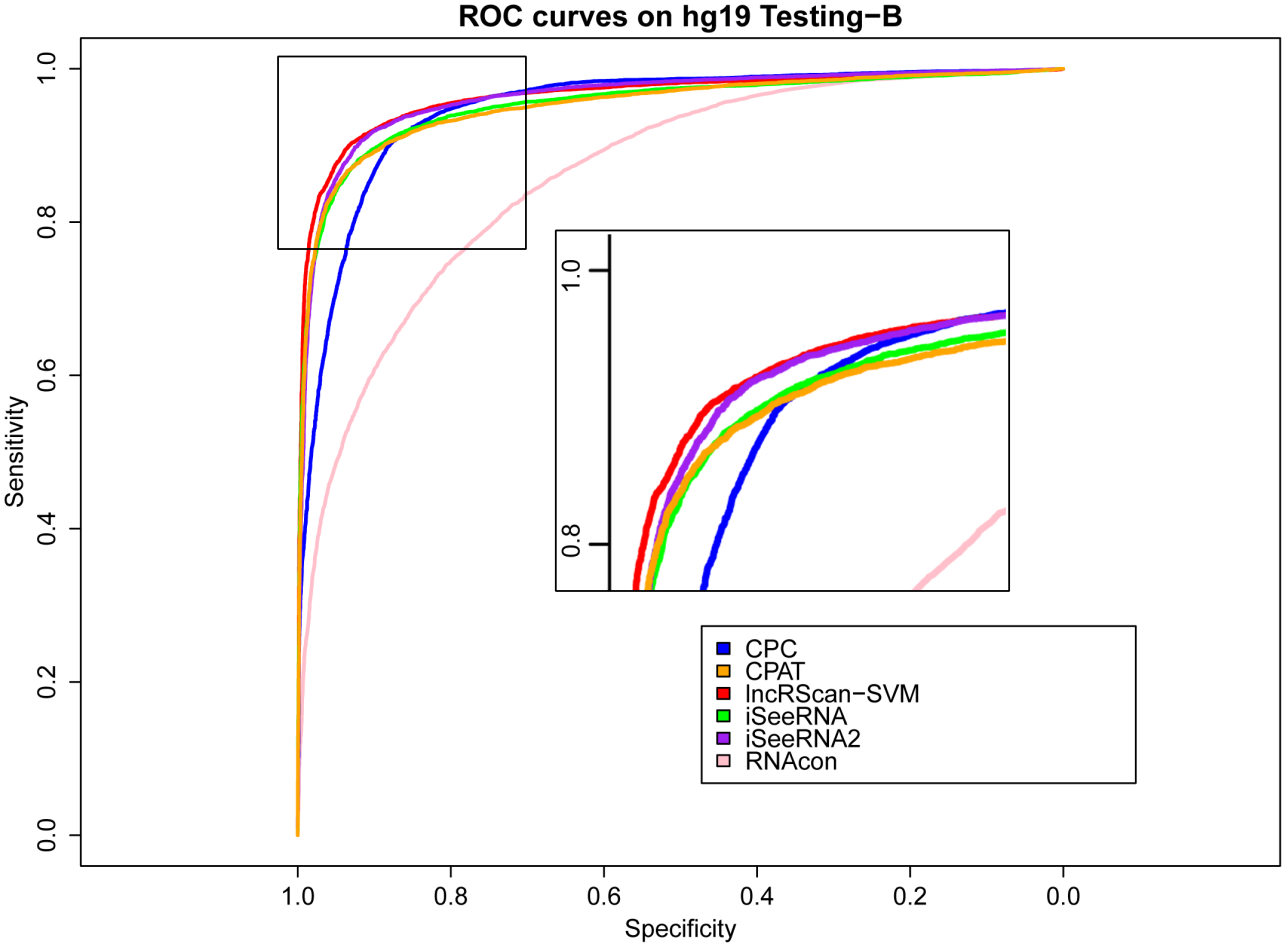
ROC curves of lncRNA prediction on hg19 Testing-B. The description of ROC curves for tests on hg19 Testing-B is the same as Fig 3.

**Fig 6:**
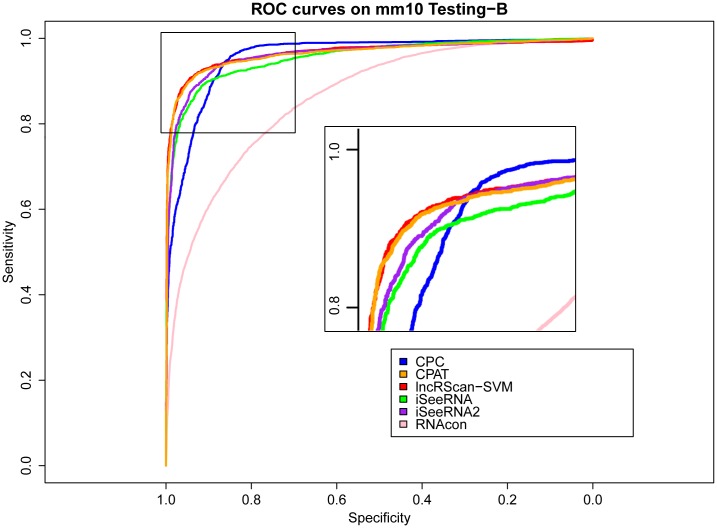
ROC curves of lncRNA prediction on mm10 Testing-B. The description of ROC curves for tests on mm10 Testing-B is the same as Fig 3.

Meanwhile, similar performance of these methods can be observed from the testing result on Testing-B of hg19 and mm10, where our lncRScan-SVM predictor consistently shows better performance than other methods. As seen from the result on mm10 Testing-B (Table 4), lncRScan-SVM is ranked the highest in almost all aspects, including SPC, ACC, MCC and AUC. As indicated by SES, CPC still has a bias towards predicting PCTs, but has poor overall performance. Although the re-trained iSeeRNA2 shows similar overall performance to lncRScan-SVM, its SPC scores are much lower than the latter one, which means iSeeRNA2 would perform less accurate for identifying lncRNAs than lncRScan-SVM. In addition, the AUC scores of these methods are visualized by ROC curves (Figs 3, 4, 5 and 6).

In addition to the accuracy evaluation, the computation time of these methods was also compared on the same platform (Linux, Ubuntu 12.04.4 LTS 64bit, 800 MHz × 4 processors and 4GB RAM). As seen from Table 5, lncRScan-SVM shows the longest computation time (Nearly twice slower than iSeeRNA, four times slower than RNAcon and ten times slower than CPAT) when conducting lncRNA prediction on Testing-A of hg19 and mm10 respectively. The computation time difference can be caused by several reasons, such as different algorithms and programming languages. As seen from the table, either of the methods with query file in FASTA, namely CPAT and RNAcon, represents shorter running time than the other ones with GTF is mainly because the latter one needs longer time to extract sequence information according to the gene annotation in GTF. CPAT is faster than RNAcon because the logistic regression model used by the former one could be faster than SVM used by RNAcon. And our lncRScan-SVM is slower than iSeeRNA is mainly because we used Python scripts to implement our method, which can be optimized and accelerated using C programming in the future. Despite of that, the computation time of current lncRScan-SVM could be acceptable since its running time for precessing thousands of transcripts is still limited in several minutes or seconds. It is worth noting that CPC was not taken into account in the comparison because it is quite slow when running on the same platform. For example, it takes about 3 minutes to process only one sequence, which is much slower than the other methods.

**Table 5:** Computation time comparison on Testing-A of hg19 and mm10.

	**query file format**	**hg19 (seconds, s)**	**mm10 (seconds, s)**
*lncRScan-SVM*	GTF	170	86
iSeeRNA	GTF	88	59
iSeeRNA2	GTF	81	59
RNAcon	FASTA	42	16
CPAT	FASTA	19	8

There are 20000 and 7000 samples in the Testing-A of hg19 and mm10 respectively.

### Prediction on known human lncRNA datasets

**Fig 7:**
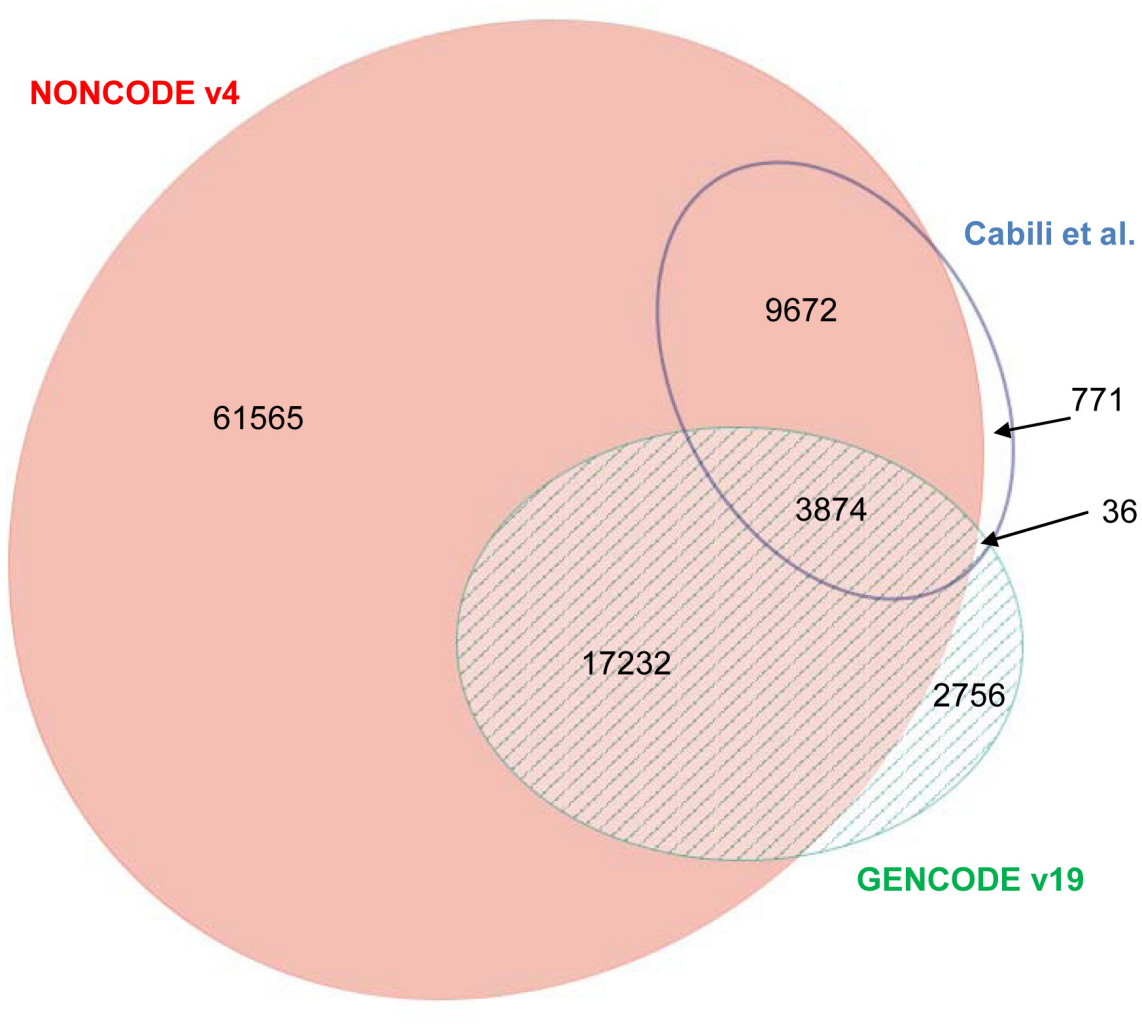
Overlap between three human lncRNA datasets. The lncRNA counts of the three human lncRNA datasets are proportional to the area of the ellipses. The largest ellipse denotes NONCODEv4 lncRNAs. The right-down and right-up ones are for GENCODEv19 and Cabili et al. lncRNAs respectively.

In addition to the high-quality GENCODE lncRNA dataset, previous studies have published several other lncRNA datasets, such as the NONCODEv4 dataset containing 92343 human lncRNAs [13] and the Cabili et al. dataset containing 14353 human long intergenic ncRNAs (lincRNAs) [7], which can be evaluated by lncRScan-SVM. Before conducting lncRNA prediction, we checked the overlap between these datasets. As seen from Fig 7 plotted by eulerAPE v3 [33], the human lncRNA annotated by NONCODEv4 contains most of the lncRNAs annotated by Cabili et al. (21106, ∼ 88.32%) and GENCODEv19 (13546, ∼ 94.38%) due to the fact that NONCODEv4 published on 2014 collects a large number of ncRNAs from various literatures and databases [13]. On the other hand, the overlapping area between Cabili et al. and GENCODEv19 lncRNAs is small (3874, ∼ 16.36% of GENCODEv19 or ∼ 27.24% of Cabili et al.).

Then we applied lncRScan-SVM to the Cabili et al. and NONCODEv4 datasets respectively, compared with other methods (See Table 6). As a result, lncRScan-SVM successfully identified 14069 (98.52%) of 14281 lincRNAs annotated by Cabili et al., which were generated by a complex filtering pipeline mentioned in their paper [7]. On the NONCODEv4 lncRNA dataset, lncRScan-SVM successfully predicted 77435 (85.63%) of the totalling 90429 lncRNAs, which is much lower than that on the Cabili et al. dataset due to the fact that the lncRScan-SVM prediction model trained on GENCODEv19 that contains 12152 (50.85%) lincRNAs might have a bias towards predicting lincRNAs rather than non-intergenic lncRNAs. To test this hypothesis, we conducted lncRScan-SVM on the 43730 lincRNAs (the lncRNAs located in the intergenic regions of GENCODEv19 protein coding transcripts) extracted from the NONCODEv4 dataset. As a result, 41984 (96.01%) lincRNA were predicted successfully. Similarly, the prediction ratios of the other methods all decline as test sets change from lncRNA to lincRNA, which means they all have a bias towards predicting lincRNAs. In contrast, most of the other tools represent lower prediction ratios than lncRScan-SVM on the three datasets except CPAT, which has the highest prediction ratio (90.29%) on NONCODEv4 lncRNA dataset since CPAT has a bias towards predicting the lncRNAs (See previous section). However, CPAT still represents poorer performance on either Cabili et al. or NONCODEv4 lincRNAs. Overall, the prediction of these methods on the general lncRNA datasets is consistent with that on our gold-standard sets.

**Table 6:** Prediction on known human lncRNA datasets.

	Human lincRNA of Cabili et al. (14281 processed/14353 total)	Human lncRNA of NONCODEv4 (90429 processed/92343 total)	Human lincRNA of NONCODEv4 (43730 processed/45644 total)
*lncRScan-SVM*	**14069 (98.52%)**	77435 (85.63%)	**41984 (96.01%)**
iSeeRNA	13887 (97.24%)	75535 (83.53%)	41143 (94.08%)
iSeeRNA2	13848 (96.97%)	73439 (81.21%)	40821 (93.35%)
RNAcon	12651 (88.59%)	67873 (75.06%)	35431 (81.02%)
CPAT	13973 (97.84%)	**81651 (90.29%)**	41276 (94.39%)
CPC	10910 (76.40%)	Not available^*a*^	26380 (60.32%)

The lncRNA prediction performance of lncRScan-SVM is compared with iSeeRNA, iSeeRNA2, RNAcon, CPAT and CPC on on three human lncRNA datasets. The biggest prediction ratio is highlighted by bold font.

^*a*^ CPC was not available for predicting NONCODEv4 human lncRNAs and reported *TOO_MANY_ILLEGAL_CHARACTERS* after submitting through its web server.

In addition, the remaining 72 lincRNAs of Cabili et al. dataset and 1964 lncRNAs or 1914 lincRNAs of NONCODEv4 were not took into account analysis because they are not annotated on the main reference chromosomes, such as chr1, chr2 and so on, but are annotated on on the other regions such as patches, scaffolds and haplotypes, and they can not be processed by several methods.

### Discussion

The lncRNA prediction problem can be solved by several computational methods mentioned above, especially our lncRScan-SVM performs better than the others in several aspects by integrating information extracted from gene structure, sequence composition and conservation in the SVM framework. However several problems remain to be specified. First, novel features and models can be developed to improve the prediction performance. For example, features can be extracted from secondary structure of RNA since functional lncRNAs may have special patterns in the secondary structure. Second, current lncRNA prediction methods present a bias towards predicting lincRNAs, so new distinguishable features should be extracted for predicting those non-intergenic lncRNAs, thereby helping solve the third problem, that is a more detailed catalogue of lncRNAs needs to be created by classifying the lncRNAs into subclasses corresponding to various functions. Last but not least, it is worth noting that classifying PCTs and LNCTs might be meaningless since some lncRNAs can be bifunctional, that is some noncoding RNAs can be translated into peptides in particular circumstances [16, 34].

Therefore, identifying PCTs or LNCTs is not only a simple classification problem, but also fundamental for further analysis, e.g. lncRNA function study. It is hopeful that various datasets and novel hypothesis can help improve the prediction accuracy and further deepen our understanding of lncRNA functions.

## Conclusions

Current lncRNA studies have been accelerated by various datasets and efficient bioinformatic tools. Here we proposed lncRScan-SVM, which performs better than several popular methods in predicting the lncRNAs. lncRScan-SVM is quite useful for lncRNA gene annotation and can be integrated into pipelines of lncRNA study.

## Supporting Information

S1 Dataset A compressed file containing GTF files of Training-A, Testing-A and Testing-B transcripts. (ZIP)

S1 File Comparison of three feature selection strategies. To select a set of good features for model training and testing, we compared three feature selection (FS) strategies.
(DOC)
