# Supplementary material for: lncRScan-SVM: A Tool for Predicting Long Non-Coding RNAs Using Support Vector Machine
Source: PLoS One. 2015 Oct 5;10(10):e0139654. doi: 10.1371/journal.pone.0139654 (PMC4593643; doi:10.1371/journal.pone.0139654)
Supplement: S1 File — To select a set of good features for model training and testing, we compared three feature selection (FS) strategies. (DOC) [file pone.0139654.s002.doc]

Comparison of three feature selection strategies

To select a set of good features for model training and testing, we compared three feature selection (FS) strategies.

1. fselect.py, a LIBSVM tool

The LIBSVM website provides a python script to use F-score for selecting features. The details of the strategy can be found in Reference [1]. In short, the strategy selects features with high F-scores and then applies SVM for training/prediction. By executing fselect.py on our training data, namely training-A, we get a list of F-scores for all candidate features (Table-S1).

Table S1 F-scores of features

| Index | feature | F-score(hg19) | F-score(mm10) |
| --- | --- | --- | --- |
| 1 | transcript length | 0.107046 | 0.046509 |
| 2 | GC content | 0.05904 | 0.024198 |
| 3 | stop codon std | 0.149335 | 0.038628 |
| 4 | CDS length | 0.289899 | 0.080757 |
| 5 | CDS percentage | 0.554848 | 0.689886 |
| 6 | CDS score | 0.273903 | 0.079497 |
| 7 | consv | 0.953318 | 0.645819 |
| 8 | exon count | 0.222105 | 0.114193 |
| 9 | exon length | 0.002628 | 0.004072 |
| 10 | ACG | 0.109817 | 0.060646 |
| 11 | CCG | 0.115708 | 0.049835 |
| 12 | CGA | 0.1692 | 0.086962 |
| 13 | CGC | 0.083588 | 0.028392 |
| 14 | CGG | 0.119105 | 0.046662 |
| 15 | CGT | 0.094812 | 0.04925 |
| 16 | GCG | 0.093874 | 0.036152 |
| 17 | TCG | 0.134927 | 0.048681 |
| 18 | CTA | 0.031324 | 0.02639 |
| 19 | GGG | 0.073896 | 0.024925 |
| 20 | GTA | 0.109957 | 0.081203 |
| 21 | TAA | 0.00237 | 0.002247 |
| 22 | TAC | 0.078689 | 0.073203 |
| 23 | TAG | 0.000823 | 0.001506 |

The F-scores of hg19 and mm10 are ranked respectively. Then, four features sizes 23 / 11 / 5 / 2 were tried in 10-fold cross-validation based on SVM respectively.

For hg19, the maximum validation accuracy is 89.2 when using the top five features ranked by F-scores, which are **CDS length(4), CDS percentage(5), CDS score(6) , consv(7)** and **exon count(8)**.

For mm10, the maximum accuracy is 89.56 when using the top 11 features ranked, which are **CDS length(4), CDS percentage(5), CDS score(6), consv(7), exon count(8), ACG(10), CCG(11), CGA(12), CGT(15) , GTA(20)** and **TAC(22)**.

1. WEKA[2] attribute selection

WEKA is a platform integrating various machine learning algorithms. We used WEKA-3.6.11 for FS. By using WEKA explorer, we first input our training-A data to WEKA. Then in “Select attributes” of the explorer, we tried different search methods for FS (Table-S2 and Table-S3). We used all training set in setting the attribute selection mode.

Table S2 WEKA FS on hg19 training-A

| Evaluator | Search method^2^ | Index of selected features | metrit |
| --- | --- | --- | --- |
| CFS^1^ | BestFirst | 5,6,7 | 0.724 |
|  | Random | 5,7,8,10,11,12,15,17,18,20,22 | 0.598 |
|  | LinearForwardSelection | 5,6,7 | 0.724 |
|  | Exhaustive | 5,6,7 | 0.724 |
|  | Scatter | 5,6,7 | 0.724 |

1 CFS - a correlation-based filter method

2 All search methods were run with default parameters provided by WEKA.

Table S3 WEKA FS on mm10 training-A

| Evaluator | Search method | Index of selected features | metrit |
| --- | --- | --- | --- |
| CFS | BestFirst | 1,5,7,20 | 0.721 |
|  | Random | 1,5,7,12,15,20,22 | 0.501 |
|  | LinearForwardSelection | 1,5,7,20 | 0.721 |
|  | Exhaustive | 1,5,7,20 | 0.721 |
|  | Scatter | 1,5,7,20 | 0.721 |

As seen from Table S2, the best feature group for hg19 includes **CDS percentage (5), CDS score (6)** and **consv (7)**. Similarly, the best feature group of WEKA includes **transcript length (1), CDS percentage (5), consv (7)** and **GTA (20)** for training mm10 SVM models.

1. Manual selection

We also conducted manual selection based on the biological meaning of the features and the output from various FS strategies. As a result, we manually selected six features, namely **transcript length (1), stop codon std (3), CDS score (6), consv (7), exon count (8) and exon length (9)**, for either hg19 or mm10.

To find out the best group of features, we compared the best groups of all three FS strategies mentioned above using indicators ACC and MCC (Table S4). As a result, the ‘Manual’ selected feature groups perform the best. Thus, the six features, namely **transcript length (1), stop codon std (3), CDS score (6), consv (7), exon count (8) and exon length (9)** were finally chose as the features of lncRScan-SVM.

Table S4 FS strategy comparison

| Species | Method | Best group of features | ACC(%) | MCC(%) |
| --- | --- | --- | --- | --- |
| hg19 | fselect.py | 4,5,**6,7,8** | 91.4 | 82.83 |
|  | WEKA | 5,6,7 | 90.6 | 81.21 |
|  | Manual | 1,3,**6,7,8**,9 | **91.54** | **83.17** |
| mm10 | fselect.py | 4,**6,7,8**,10,11,12,15,20,22 | 91.914 | 84.02 |
|  | WEKA | 1,5,7,20 | 91.171 | 82.41 |
|  | Manual | 1,3,**6,7,8**,9 | **92.214** | **84.59** |

References

[1] Y.-W. Chen and C.-J. Lin, Combining SVMs with various feature selection strategies, http://www.csie.ntu.edu.tw/~cjlin/papers/features.pdf

[2] Mark Hall, Eibe Frank, Geoffrey Holmes, Bernhard Pfahringer, Peter Reutemann, Ian H. Witten (2009); The WEKA Data Mining Software: An Update; SIGKDD Explorations, Volume 11, Issue 1.
